# Bis(l-serinium) oxalate dihydrate: polymorph II

**DOI:** 10.1107/S160053681302727X

**Published:** 2013-10-19

**Authors:** Marta Kulik, Aleksandra Pazio, Krzysztof Wozniak

**Affiliations:** a Chemistry Department, University of Warsaw, Pasteura 1, 02-093 Warszawa, Poland, and, Centre of New Technologies, University of Warsaw, Zwirki i Wigury 93, 02-089 Warszawa, Poland; b Chemistry Department, University of Warsaw, Pasteura 1, 02-093 Warszawa, Poland

## Abstract

A corrected and improved structure of the polymorph II of 2C_3_H_8_NO_3_^+^·C_2_O_4_^2−^·2H_2_O, based on single-crystal data, is presented. The structure is refined with anisotropic displacement parameters for all non-H atoms and all H atoms are located. Due to the charged moieties, the structure is classified as a mol­ecular salt. Inter­molecular O—H⋯O^−^, O—H⋯O and N^+^—H⋯O^−^hydrogen bonds link the components of the structure. The l-serinium cations and oxalate anions form a network of channels in [100] direction, filled with the water molecules of crystallization. The dihedral angle between the CO_2_ units of the oxalate dianion is 10.2 (3)°

## Related literature

Crystallization of serine with oxalic acid leads to diverse mol­ecular salts, with some of them exhibiting polymorphism. The polymorphs I and II of 2C_3_H_7_NO_3_^+^·C_2_O_4_^2−^·2H_2_O have already been described, see: Braga *et al.* (2013 ▶). Form II was determined by powder X-ray diffraction methods and therefore the crystal structure lacks properly located H atoms and anisotropic displacement parameters of all heavy atoms in the structure.
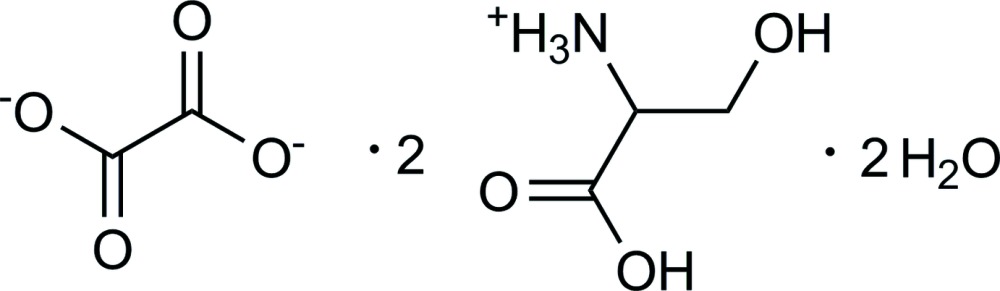


## Experimental

### 

#### Crystal data

2C_3_H_8_NO_3_^+^·C_2_O_4_^2−^·2H_2_O
*M* *_r_* = 336.26
Monoclinic, 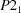

*a* = 5.1524 (2) Å
*b* = 11.1467 (4) Å
*c* = 12.4478 (5) Å
β = 99.967 (4)°
*V* = 704.12 (5) Å^3^
*Z* = 2
Mo *K*α radiation
μ = 0.15 mm^−1^
*T* = 90 K
0.28 × 0.10 × 0.08 mm

#### Data collection

Agilent Xcalibur Opal diffractometer
Absorption correction: multi-scan [*SCALE3 ABSPACK* (Blessing, 1995 ▶) and *CrysAlis PRO* (Agilent, 2012 ▶)] *T*_min_ = 0.980, *T*_max_ = 1.000
17202 measured reflections
2876 independent reflections
2430 reflections with *I* > 2σ(*I*)
*R*_int_ = 0.030

#### Refinement

*R*[*F* ^2^ > 2σ(*F* ^2^)] = 0.032
*wR*(*F* ^2^) = 0.065
*S* = 1.01
2876 reflections
217 parameters
5 restraints
H atoms treated by a mixture of independent and constrained refinement
Δρ_max_ = 0.17 e Å^−3^
Δρ_min_ = −0.17 e Å^−3^

### 

Data collection: *CrysAlis PRO* (Agilent, 2012 ▶); cell refinement: *CrysAlis PRO*; data reduction: *CrysAlis PRO* and *SORTAV* (Blessing, 1995 ▶); program(s) used to solve structure: *SHELXS97* (Sheldrick, 2008 ▶); program(s) used to refine structure: *SHELXL97* (Sheldrick, 2008 ▶); molecular graphics: *ORTEP-3 for Windows* (Farrugia, 2012 ▶) and *DIAMOND* (Brandenburg, 1999 ▶); software used to prepare material for publication: *SHELXL97*.

## Supplementary Material

Crystal structure: contains datablock(s) I. DOI: 10.1107/S160053681302727X/gk2581sup1.cif

Structure factors: contains datablock(s) I. DOI: 10.1107/S160053681302727X/gk2581Isup2.hkl

Supplementary material file. DOI: 10.1107/S160053681302727X/gk2581Isup3.cdx

Supplementary material file. DOI: 10.1107/S160053681302727X/gk2581Isup4.cml

Additional supplementary materials:  crystallographic information; 3D view; checkCIF report

## Figures and Tables

**Table 1:** Hydrogen-bond geometry (Å, °)

*D*—H⋯*A*	*D*—H	H⋯*A*	*D*⋯*A*	*D*—H⋯*A*
N1—H1*A*⋯O9^i^	0.89	1.94	2.826 (2)	172
N1—H1*B*⋯O4^ii^	0.89	2.00	2.809 (2)	151
N1—H1*C*⋯O6^iii^	0.89	1.99	2.779 (2)	148
N1—H1*C*⋯O4^iii^	0.89	2.34	3.044 (2)	136
N2—H2*A*⋯O7	0.89	2.14	3.020 (2)	172
N2—H2*A*⋯O5	0.89	2.66	3.200 (2)	120
N2—H2*B*⋯O7^iv^	0.89	2.04	2.922 (2)	169
N2—H2*C*⋯O8^ii^	0.89	1.93	2.811 (2)	168
O2—H2*D*⋯O6^iv^	0.82	1.69	2.5122 (19)	175
O3—H3⋯O4^iii^	0.82	1.98	2.7887 (18)	168
O8—H8*A*⋯O9^i^	0.84 (1)	2.08 (1)	2.910 (2)	174 (2)
O8—H8*B*⋯O13^v^	0.84 (1)	1.90 (1)	2.730 (2)	167 (2)
O9—H9*A*⋯O8	0.84 (1)	2.07 (1)	2.911 (2)	175 (2)
O9—H9*B*⋯O3^vi^	0.84 (1)	1.94 (1)	2.7607 (19)	164 (2)
O12—H12*A*⋯O5^vii^	0.82	1.73	2.5513 (19)	177
O13—H13⋯O5	0.82	1.98	2.7637 (19)	159
O13—H13⋯O7	0.82	2.55	3.0521 (19)	121

Symmetry codes: (i) 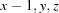; (ii) 
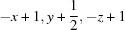
; (iii) 
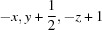
; (iv) 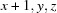; (v) 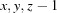; (vi) 
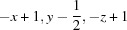
; (vii) 
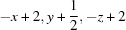
.

**Fig. 1.**
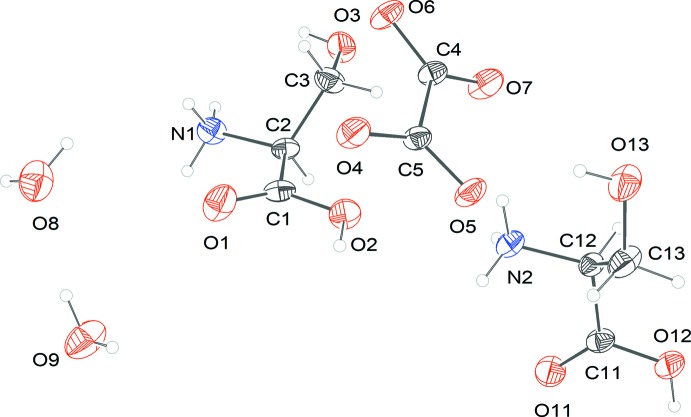
Atomic displacement parameters at the 50% probability level and atom labeling scheme of the asymmetric part of the unit cell in polymorph II

**Fig. 2.**
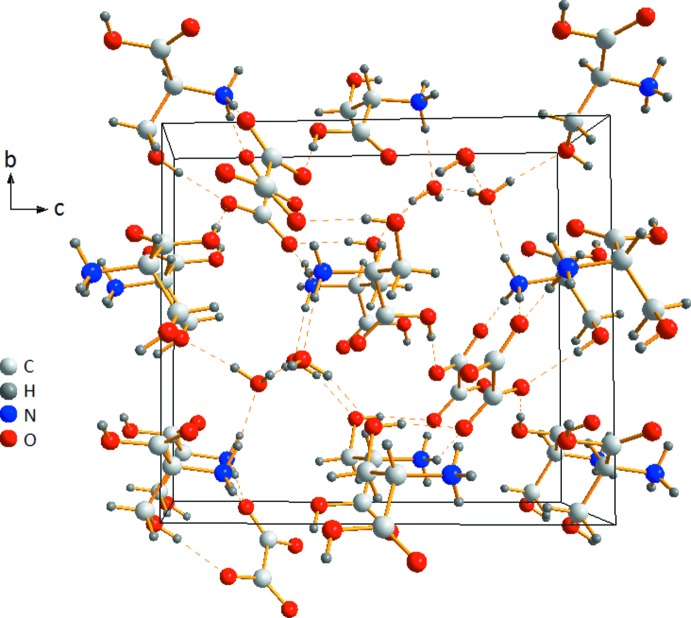
The crystal packing in polymorph II viewed along the a axis. Dashed lines indicate hydrogen bonds.

## supplementary crystallographic information

### 1. Comment

Divers crystal forms of molecular salts of *L*-serine with oxalic acid can be obtained by grinding or kneading powders of both compounds, as decribed earlier (Braga *et al.*, 2013). However, previously reported structure of form II of 2C_3_H_7_NO_3_^+^.C_2_O_4_^2-^.2H_2_O was based on powder diffraction X-ray data and therefore hydrogen atoms were positioned geometrically (including –NH_3_^+^ and –COOH groups) and the structure refined with isotropic displacement parameters for for L-serinium cations. Moreover, the H atoms of water molecules were not located. Crystallization of the polymorph II of 2C_3_H_7_NO_3_^+^.C_2_O_4_^2-^.2H_2_O is also possible by slow evaporation from water solution and it results in crystals of the size sufficient to perform single-crystal X-ray diffraction experiment. Hence, the proper H-atom positions can be found. Both hydrate polymorphs have different unit-cell dimensions, whereas crystal packing remains virtually very similar, with a characteristic motif of the zigzag chains formed by hydrogen bonds between the water molecules along the [100] direction. The location of hydrogen bond network surrounding the oxalate anion allows for discrimination between the polymorphic forms. The form I contains 8 hydrogen bonds between the oxalate anion and 6 neighbouring serine cations. In the polymorph II, obtained from powder data, due to the lack of proper positions of H-atoms, one can suspect the presence of 11 hydrogen bonds around the oxalate anion, located within the donor-acceptor distance in the range from 2.4 to 3.0 Å. In the polymorph II structure derived from single-crystal X-ray diffraction data, 9 hydrogen bonds are formed between the oxalate anion to the 6 neighbouring serine cations. This difference results from a wrong assignment of the carboxylic H-atom in one of the serinium cations in the structure obtained from powder data (see Fig.1, atoms O2 and O12).Both structures of the form II have different distances between the oxalate anions. The structure of Braga *et al.* (2013) presents a denser arrangement between the oxalate anions. The distances between oxygen atoms from neighbouring anions for the structure obtained by single crystal X-ray measurement range from 3.237 (2) to 4.975 (2) Å. The corresponding values in the structure derived from the powder data are between 2.63 (1) to 4.97 (1) Å.

To compare the unit cell parameters obtained by powder methods at room temperature [a= 12.5711 (6), b= 11.2144 (5), c= 5.2079 (2) Å, β= 100.529 (3)°] these parameters were also determined at room temperature from a single crystal [a= 5.1869 (2), b= 11.1906 (5), c= 12.5305 (5) Å, β= 100.485 (4)°].

### 2. Experimental

A mixture of *L*-serine (0.158 mg) with oxalic acid (0.068 mg) was dissolved in water (15 mL) in the 2:1 stoichiometric ratio and set aside to crystallize by slow evaporation at 309 K. Little needle-shaped crystals of bis(*L*-serinium) oxalate dihydrate form II (m.p. 357 K) have grown on bigger needle-shaped crystals of oxalic acid.

### 3. Refinement

All H-atoms bound to C were placed at the calculated positions and were treated as riding on the parent atom with *U*_iso_(H) = 1.2*U*_eq_(C) and C–H distances of 0.98 Å for methine and 0.97 Å for methylene groups. N^+^-bound H atoms were placed in locations indicated by a difference Fourier synthesis and were refined using a riding model, with *U*_iso_(H) = 1.5*U*_eq_(C) and N– H distance of 0.89 Å. H atoms attached to O were placed in locations indicated by a difference Fourier synthesis and were refined using a riding model with *U*_iso_(H) values set at 1.5 *U*_eq_(O) and with a distance restraint of O–H = 0.82 Å, except for the water molecules for which O-H distances were constrained to 0.840 (5) Å. An absolute structure has been assigned by reference to an unchanging chiral centre in the crystallization procedure. For the refinement 1367 Friedel pairs were not merged.

### Crystal data

**Table:**

2C_3_H_8_NO_3_^+^·C_2_O_4_^2^^−^·2H_2_O	*F*(000) = 356
*M**_r_* = 336.26	*D*_x_ = 1.586 Mg m^−^^3^
Monoclinic, *P*2_1_	Melting point: 357 K
Hall symbol: P 2yb	Mo *K*α radiation, λ = 0.71073 Å
*a* = 5.1524 (2) Å	Cell parameters from 8206 reflections
*b* = 11.1467 (4) Å	θ = 1.7–26.4°
*c* = 12.4478 (5) Å	µ = 0.15 mm^−^^1^
β = 99.967 (4)°	*T* = 90 K
*V* = 704.12 (5) Å^3^	Needle, colourless
*Z* = 2	0.28 × 0.10 × 0.08 mm

### Data collection

**Table:**

Agilent Xcalibur Opal diffractometer	2876 independent reflections
Radiation source: Enhance (Mo) X-ray Source	2430 reflections with *I* > 2σ(*I*)
Graphite monochromator	*R*_int_ = 0.030
Detector resolution: 8.4441 pixels mm^-1^	θ_max_ = 26.4°, θ_min_ = 1.7°
ω scans	*h* = −6→6
Absorption correction: multi-scan [SCALE3 ABSPACK (Blessing, 1995) and *CrysAlis PRO* (Agilent, 2012)]	*k* = −13→13
*T*_min_ = 0.980, *T*_max_ = 1.000	*l* = −15→15
17202 measured reflections	

### Refinement

**Table:**

Refinement on *F*^2^	0 constraints
Least-squares matrix: full	H atoms treated by a mixture of independent and constrained refinement
*R*[*F*^2^ > 2σ(*F*^2^)] = 0.032	*w* = 1/[σ^2^(*F*_o_^2^) + (0.0288*P*)^2^] where *P* = (*F*_o_^2^ + 2*F*_c_^2^)/3
*wR*(*F*^2^) = 0.065	(Δ/σ)_max_ < 0.001
*S* = 1.01	Δρ_max_ = 0.17 e Å^−^^3^
2876 reflections	Δρ_min_ = −0.17 e Å^−^^3^
217 parameters	Absolute structure: Flack (1983), 1367 Friedel pairs
5 restraints	Absolute structure parameter: −0.4 (9)

### Special details

**Table:**

Geometry. All esds (except the esd in the dihedral angle between two l.s. planes) are estimated using the full covariance matrix. The cell esds are taken into account individually in the estimation of esds in distances, angles and torsion angles; correlations between esds in cell parameters are only used when they are defined by crystal symmetry. An approximate (isotropic) treatment of cell esds is used for estimating esds involving l.s. planes.
Refinement. Refinement of *F*^2^ against ALL reflections. The weighted *R*-factor *wR* and goodness of fit *S* are based on *F*^2^, conventional *R*-factors *R* are based on *F*, with *F* set to zero for negative *F*^2^. The threshold expression of *F*^2^ > 2σ(*F*^2^) is used only for calculating *R*-factors(gt) *etc*. and is not relevant to the choice of reflections for refinement. *R*-factors based on *F*^2^ are statistically about twice as large as those based on *F*, and *R*- factors based on ALL data will be even larger.

### Fractional atomic coordinates and isotropic or equivalent isotropic displacement parameters (Å^2^)

**Table:**

	*x*	*y*	*z*	*U*_iso_*/*U*_eq_	
C1	0.4625 (4)	0.52285 (19)	0.52693 (16)	0.0118 (4)	
C2	0.2932 (4)	0.63226 (18)	0.49184 (14)	0.0116 (4)	
H2	0.3931	0.7053	0.5150	0.014*	
C3	0.0458 (4)	0.62912 (19)	0.54164 (15)	0.0130 (4)	
H3A	−0.0591	0.5602	0.5133	0.016*	
H3B	0.0944	0.6192	0.6200	0.016*	
C4	0.0979 (4)	0.39834 (17)	0.75125 (15)	0.0090 (4)	
C5	0.3329 (4)	0.30988 (18)	0.76828 (15)	0.0091 (4)	
C11	0.9862 (4)	0.69260 (18)	1.00499 (16)	0.0110 (4)	
C12	0.7188 (4)	0.63044 (18)	0.98188 (14)	0.0099 (4)	
H12	0.5875	0.6845	1.0039	0.012*	
C13	0.7174 (4)	0.51374 (18)	1.04585 (16)	0.0127 (4)	
H13A	0.8354	0.4565	1.0207	0.015*	
H13B	0.7810	0.5290	1.1226	0.015*	
N1	0.2229 (3)	0.62971 (16)	0.37022 (12)	0.0123 (4)	
H1A	0.1372	0.5620	0.3493	0.015*	
H1B	0.3692	0.6334	0.3414	0.015*	
H1C	0.1204	0.6922	0.3475	0.015*	
N2	0.6468 (3)	0.60909 (15)	0.86212 (12)	0.0111 (4)	
H2A	0.4974	0.5674	0.8483	0.013*	
H2B	0.7747	0.5679	0.8391	0.013*	
H2C	0.6253	0.6792	0.8274	0.013*	
O1	0.5153 (3)	0.44889 (13)	0.46302 (11)	0.0160 (3)	
O2	0.5432 (3)	0.51993 (13)	0.63342 (10)	0.0136 (3)	
H2D	0.6541	0.4670	0.6485	0.020*	
O3	−0.1098 (3)	0.73504 (12)	0.51957 (10)	0.0145 (3)	
H3	−0.1608	0.7412	0.4537	0.022*	
O4	0.3329 (3)	0.22639 (12)	0.70100 (10)	0.0127 (3)	
O5	0.5089 (3)	0.32894 (12)	0.85114 (10)	0.0118 (3)	
O6	−0.0945 (3)	0.36793 (12)	0.67897 (10)	0.0113 (3)	
O7	0.1134 (3)	0.49023 (12)	0.80848 (11)	0.0131 (3)	
O9	0.9872 (3)	0.40357 (13)	0.31699 (11)	0.0172 (3)	
O8	0.4472 (3)	0.34223 (13)	0.22167 (11)	0.0154 (3)	
O11	1.0996 (3)	0.72817 (12)	0.93378 (11)	0.0139 (3)	
O12	1.0685 (3)	0.70526 (12)	1.11088 (10)	0.0134 (3)	
H12A	1.2031	0.7460	1.1213	0.020*	
O13	0.4594 (3)	0.46436 (13)	1.03248 (11)	0.0151 (3)	
H13	0.4326	0.4231	0.9771	0.023*	
H9A	0.8281 (17)	0.388 (2)	0.2924 (17)	0.023*	
H9B	1.028 (4)	0.3640 (18)	0.3750 (11)	0.023*	
H8A	0.316 (3)	0.365 (2)	0.2477 (18)	0.023*	
H8B	0.428 (5)	0.3845 (18)	0.1648 (12)	0.023*	

### Atomic displacement parameters (Å^2^)

**Table:**

	*U*^11^	*U*^22^	*U*^33^	*U*^12^	*U*^13^	*U*^23^
C1	0.0076 (10)	0.0142 (11)	0.0139 (11)	−0.0019 (9)	0.0027 (8)	0.0010 (9)
C2	0.0106 (11)	0.0128 (10)	0.0109 (10)	0.0007 (9)	0.0004 (8)	0.0002 (9)
C3	0.0132 (11)	0.0128 (10)	0.0129 (10)	0.0019 (9)	0.0021 (9)	0.0006 (9)
C4	0.0111 (11)	0.0082 (10)	0.0078 (10)	−0.0013 (9)	0.0025 (8)	0.0036 (8)
C5	0.0070 (10)	0.0105 (10)	0.0105 (10)	−0.0028 (8)	0.0036 (8)	0.0029 (8)
C11	0.0113 (10)	0.0064 (10)	0.0148 (10)	0.0020 (8)	0.0004 (8)	−0.0018 (8)
C12	0.0104 (10)	0.0133 (10)	0.0057 (9)	0.0009 (8)	0.0011 (8)	−0.0010 (8)
C13	0.0118 (11)	0.0136 (11)	0.0117 (10)	−0.0039 (9)	−0.0008 (8)	0.0014 (8)
N1	0.0112 (9)	0.0135 (9)	0.0121 (8)	0.0019 (7)	0.0018 (7)	0.0036 (7)
N2	0.0125 (9)	0.0099 (9)	0.0100 (8)	−0.0020 (7)	−0.0001 (7)	−0.0008 (7)
O1	0.0159 (8)	0.0155 (8)	0.0149 (8)	0.0046 (7)	−0.0022 (6)	−0.0025 (6)
O2	0.0128 (8)	0.0165 (8)	0.0107 (8)	0.0072 (6)	0.0002 (6)	0.0024 (6)
O3	0.0160 (8)	0.0160 (8)	0.0108 (7)	0.0071 (6)	0.0003 (6)	0.0004 (6)
O4	0.0137 (7)	0.0114 (7)	0.0126 (7)	0.0015 (6)	0.0013 (6)	−0.0042 (6)
O5	0.0094 (7)	0.0144 (8)	0.0107 (7)	0.0015 (6)	−0.0009 (6)	−0.0009 (6)
O6	0.0114 (7)	0.0102 (7)	0.0107 (7)	0.0012 (6)	−0.0023 (6)	−0.0009 (5)
O7	0.0119 (7)	0.0123 (8)	0.0143 (7)	0.0014 (6)	0.0004 (6)	−0.0034 (6)
O9	0.0154 (8)	0.0188 (9)	0.0160 (8)	−0.0037 (7)	−0.0017 (7)	0.0029 (7)
O8	0.0183 (8)	0.0149 (8)	0.0137 (8)	0.0000 (7)	0.0051 (7)	0.0014 (6)
O11	0.0138 (7)	0.0161 (7)	0.0124 (7)	−0.0030 (6)	0.0036 (6)	−0.0001 (6)
O12	0.0092 (7)	0.0192 (8)	0.0117 (7)	−0.0074 (6)	0.0013 (5)	−0.0038 (6)
O13	0.0171 (8)	0.0161 (8)	0.0113 (7)	−0.0083 (6)	0.0004 (6)	−0.0023 (6)

### Geometric parameters (Å, º)

**Table:**

C1—O1	1.209 (2)	C12—H12	0.9800
C1—O2	1.319 (2)	C13—O13	1.422 (2)
C1—C2	1.519 (3)	C13—H13A	0.9700
C2—N1	1.495 (2)	C13—H13B	0.9700
C2—C3	1.511 (3)	N1—H1A	0.8900
C2—H2	0.9800	N1—H1B	0.8900
C3—O3	1.427 (2)	N1—H1C	0.8900
C3—H3A	0.9700	N2—H2A	0.8900
C3—H3B	0.9700	N2—H2B	0.8900
C4—O7	1.242 (2)	N2—H2C	0.8900
C4—O6	1.264 (2)	O2—H2D	0.8200
C4—C5	1.547 (3)	O3—H3	0.8200
C5—O4	1.252 (2)	O9—H9A	0.843 (5)
C5—O5	1.268 (2)	O9—H9B	0.841 (5)
C11—O11	1.210 (2)	O8—H8A	0.838 (5)
C11—O12	1.321 (2)	O8—H8B	0.842 (5)
C11—C12	1.524 (3)	O12—H12A	0.8200
C12—N2	1.492 (2)	O13—H13	0.8200
C12—C13	1.526 (3)		
			
O1—C1—O2	125.23 (19)	N2—C12—H12	108.2
O1—C1—C2	122.80 (17)	C11—C12—H12	108.2
O2—C1—C2	111.96 (17)	C13—C12—H12	108.2
N1—C2—C3	109.96 (16)	O13—C13—C12	110.96 (16)
N1—C2—C1	107.60 (15)	O13—C13—H13A	109.4
C3—C2—C1	110.29 (17)	C12—C13—H13A	109.4
N1—C2—H2	109.7	O13—C13—H13B	109.4
C3—C2—H2	109.7	C12—C13—H13B	109.4
C1—C2—H2	109.7	H13A—C13—H13B	108.0
O3—C3—C2	112.81 (17)	C2—N1—H1A	109.5
O3—C3—H3A	109.0	C2—N1—H1B	109.5
C2—C3—H3A	109.0	H1A—N1—H1B	109.5
O3—C3—H3B	109.0	C2—N1—H1C	109.5
C2—C3—H3B	109.0	H1A—N1—H1C	109.5
H3A—C3—H3B	107.8	H1B—N1—H1C	109.5
O7—C4—O6	126.43 (19)	C12—N2—H2A	109.5
O7—C4—C5	118.41 (16)	C12—N2—H2B	109.5
O6—C4—C5	115.16 (16)	H2A—N2—H2B	109.5
O4—C5—O5	125.86 (18)	C12—N2—H2C	109.5
O4—C5—C4	118.22 (16)	H2A—N2—H2C	109.5
O5—C5—C4	115.92 (16)	H2B—N2—H2C	109.5
O11—C11—O12	125.74 (19)	C1—O2—H2D	109.5
O11—C11—C12	123.09 (18)	C3—O3—H3	109.5
O12—C11—C12	111.14 (16)	H9A—O9—H9B	107 (2)
N2—C12—C11	108.76 (15)	H8A—O8—H8B	100 (2)
N2—C12—C13	111.26 (16)	C11—O12—H12A	109.5
C11—C12—C13	112.01 (16)	C13—O13—H13	109.5
			
O1—C1—C2—N1	−2.1 (3)	O7—C4—C5—O5	10.2 (2)
O2—C1—C2—N1	177.53 (16)	O6—C4—C5—O5	−169.46 (16)
O1—C1—C2—C3	117.8 (2)	O11—C11—C12—N2	7.0 (3)
O2—C1—C2—C3	−62.5 (2)	O12—C11—C12—N2	−175.16 (15)
N1—C2—C3—O3	−66.8 (2)	O11—C11—C12—C13	130.4 (2)
C1—C2—C3—O3	174.68 (15)	O12—C11—C12—C13	−51.8 (2)
O7—C4—C5—O4	−170.34 (17)	N2—C12—C13—O13	−64.5 (2)
O6—C4—C5—O4	10.0 (2)	C11—C12—C13—O13	173.51 (15)

### Hydrogen-bond geometry (Å, º)

**Table:**

*D*—H···*A*	*D*—H	H···*A*	*D*···*A*	*D*—H···*A*
N1—H1*A*···O9^i^	0.89	1.94	2.826 (2)	172
N1—H1*B*···O4^ii^	0.89	2.00	2.809 (2)	151
N1—H1*C*···O6^iii^	0.89	1.99	2.779 (2)	148
N1—H1*C*···O4^iii^	0.89	2.34	3.044 (2)	136
N2—H2*A*···O7	0.89	2.14	3.020 (2)	172
N2—H2*A*···O5	0.89	2.66	3.200 (2)	120
N2—H2*B*···O7^iv^	0.89	2.04	2.922 (2)	169
N2—H2*C*···O8^ii^	0.89	1.93	2.811 (2)	168
O2—H2*D*···O6^iv^	0.82	1.69	2.5122 (19)	175
O3—H3···O4^iii^	0.82	1.98	2.7887 (18)	168
O8—H8*A*···O9^i^	0.84 (1)	2.08 (1)	2.910 (2)	174 (2)
O8—H8*B*···O13^v^	0.84 (1)	1.90 (1)	2.730 (2)	167 (2)
O9—H9*A*···O8	0.84 (1)	2.07 (1)	2.911 (2)	175 (2)
O9—H9*B*···O3^vi^	0.84 (1)	1.94 (1)	2.7607 (19)	164 (2)
O12—H12*A*···O5^vii^	0.82	1.73	2.5513 (19)	177
O13—H13···O5	0.82	1.98	2.7637 (19)	159
O13—H13···O7	0.82	2.55	3.0521 (19)	121

Symmetry codes: (i) *x*−1, *y*, *z*; (ii) −*x*+1, *y*+1/2, −*z*+1; (iii) −*x*, *y*+1/2, −*z*+1; (iv) *x*+1, *y*, *z*; (v) *x*, *y*, *z*−1; (vi) −*x*+1, *y*−1/2, −*z*+1; (vii) −*x*+2, *y*+1/2, −*z*+2.
